# Mobile health strategies for blood pressure self-management in urban populations with digital barriers: systematic review and meta-analyses

**DOI:** 10.1038/s41746-021-00486-5

**Published:** 2021-07-22

**Authors:** Elaine C. Khoong, Kristan Olazo, Natalie A. Rivadeneira, Sneha Thatipelli, Jill Barr-Walker, Valy Fontil, Courtney R. Lyles, Urmimala Sarkar

**Affiliations:** 1 Division of General Internal Medicine at Zuckerberg San Francisco General Hospital, Department of Medicine, University of California San Francisco, San Francisco, CA USA; 2 UCSF Center for Vulnerable Populations at Zuckerberg San Francisco General Hospital, San Francisco, CA USA; 3 Department of Medicine, Northwestern University, Evanston, IL USA; 4 Zuckerberg San Francisco General Hospital Library, University of California San Francisco, San Francisco, CA USA

**Keywords:** Patient education, Hypertension, Outcomes research, Health services

## Abstract

Mobile health (mHealth) technologies improve hypertension outcomes, but it is unknown if this benefit applies to all populations. This review aimed to describe the impact of mHealth interventions on blood pressure outcomes in populations with disparities in digital health use. We conducted a systematic search to identify studies with systolic blood pressure (SBP) outcomes located in urban settings in high-income countries that included a digital health disparity population, defined as mean age ≥65 years; lower educational attainment (≥60% ≤high school education); and/or racial/ethnic minority (<50% non-Hispanic White for US studies). Interventions were categorized using an established self-management taxonomy. We conducted a narrative synthesis; among randomized clinical trials (RCTs) with a six-month SBP outcome, we conducted random-effects meta-analyses. Twenty-nine articles (representing 25 studies) were included, of which 15 were RCTs. Fifteen studies used text messaging; twelve used mobile applications. Studies were included based on race/ethnicity (14), education (10), and/or age (6). Common intervention components were: lifestyle advice (20); provision of self-monitoring equipment (17); and training on digital device use (15). In the meta-analyses of seven RCTs, SBP reduction at 6-months in the intervention group (mean SBP difference = −4.10, 95% CI: [−6.38, −1.83]) was significant, but there was no significant difference in SBP change between the intervention and control groups (*p* = 0.48). The use of mHealth tools has shown promise for chronic disease management but few studies have included older, limited educational attainment, or minority populations. Additional robust studies with these populations are needed to determine what interventions work best for diverse hypertensive patients.

## Introduction

Mobile health (mHealth) refers to the use of mobile devices (e.g., mobile phones, tablets) to achieve health objectives^1^. Numerous mHealth applications for chronic disease management have been developed to enable the provision of care outside a traditional clinical setting^2^. Some studies have shown mobile health strategies to be effective for the management of chronic diseases including diabetes, asthma, and cardiovascular disease^3–5^. However, the adoption and efficacy of mHealth for chronic disease management may vary by population groups.

In particular, studies have shown that individuals who are older, have lower educational attainment, or identify as persons of color are less likely to use mHealth tools, potentially due to challenges related to digital literacy or poor usability of current digital health tools^6–12^. Moreover, few studies have investigated the utility of mHealth in these populations in high-income countries such as the United States^13–16^. Over 85% of non-White and 80% of low-income American adults use the Internet, including two-thirds of adults over 65^17^. These groups are not only interested in using mHealth to improve their health but may be particularly suited to mHealth interventions since they are more likely to access the Internet exclusively using mobile devices^11,17,18^. These populations often experience cost-related barriers to care and difficulties attending in-person office visits, whether due to mobility or time limitations; both barriers can potentially be addressed by mHealth strategies^19,20^.

In particular, hypertension is a highly prevalent disease, affecting one in three adults^21^, that despite the relative ease of treatment has disparities based on educational attainment, race/ethnicity, and age^21–23^. In US adults, Hispanic and Black populations have lower rates of hypertension control than non-Hispanic White populations^21^. Data also suggest that nearly one-third of adults 60+ years old not on anti-hypertensive medications have hypertension. Similarly, among adults with less than high school education, 11% have hypertension versus 8% in the college-educated population^24^.

Given that contributors to disparities in hypertension outcomes include cost and inadequate use of primary care, mHealth solutions could play a crucial role in addressing disparities^25,26^. Relative to other chronic diseases, hypertension is well suited to remote management, and previous reviews have shown telemonitoring and interactive voice response to be effective for hypertension management^27–30^., However, the authors are not aware of any reviews focused on newer mobile technologies (short messaging service, apps, tablets) for self-management of blood pressure in populations (such as lower educational attainment, minority, or older populations) that face barriers accessing care and using digital health tools. Prior reviews about the effectiveness of mHealth tools for blood pressure self-management^31,32^ have not specifically focused on these populations, which are often poorly represented in digital health studies^33^. Therefore, we conducted this review as a first step to understanding the efficacy of mobile health use in blood pressure management for patients that have lower educational attainment, are older, or are persons of color.

## Results

**Fig. 1:**
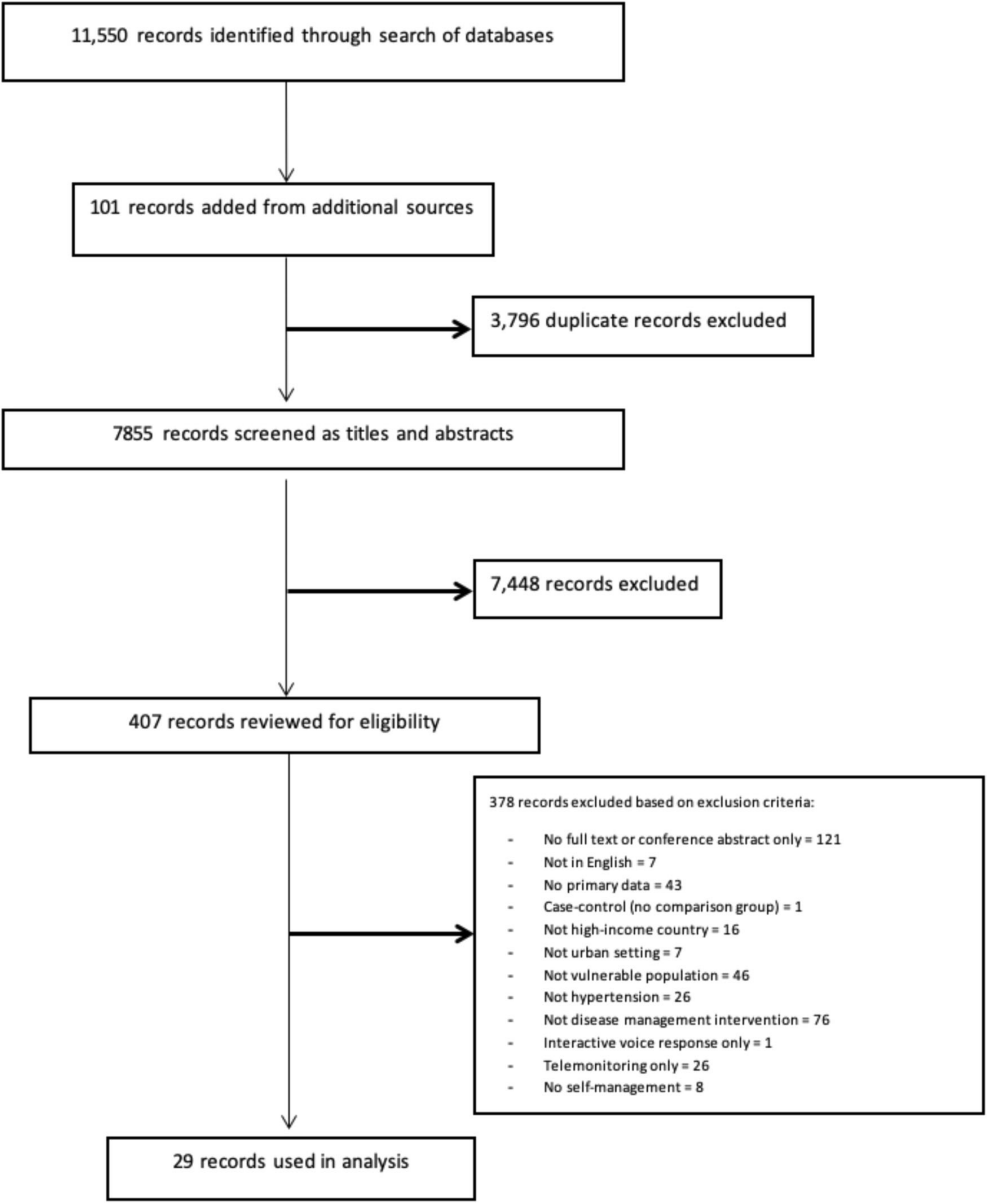
PRISMA flowchart of studies included in the review. This flowchart shows the number of records identified from the search (7855 non-duplicative records), the number of records excluded based on title and abstract (7448), and the number of studies excluded based on full article review (378), and the reasons for exclusions. Twenty-nine research articles (about 25 studies) were included in the analysis.

The literature search and subsequent search update identified 11,550 articles. After excluding duplicates and identifying articles through additional sources, we screened 7855 articles for inclusion based on title and abstract. We assessed the full text of 407 articles for eligibility and eliminated 378 based on previously established exclusion criteria. Twenty-nine articles^34–62^ were included in the final analysis as indicated by the Preferred Reporting Items for Systematic Reviews and Meta-Analyses (PRISMA) chart (Fig. 1), which represented a total of 25 different studies.

### Characteristics and participant traits in included studies

Table 1 presents the characteristics of the 25 studies (grouped by study design). Sixteen studies were conducted in the United States, three from Spain, and one each from Australia, Germany, South Korea, United Kingdom, Hong Kong, or Chile.

**Table 1:** Study design, quality, and participant characteristics of included studies.

Author and year	Location	Duration and design	Pilot study	Sample size (*N*)	Age (mean)	Racial/ethnic minority (%)	≤HS educ. (%)	GRADE quality
Randomized clinical trials								
Alonso-Domínguez, 2019	Salamanca, Spain	12-mo RCT		408	60.6	Non-US study	**83.33%**	Moderate
Buis, 2017	Detroit, MI	1-mo RCT	X	123	49	**100% Black**	49.10%	Moderate-low
Chow, 2015	Sydney, Australia	6-mo RCT		710	57.6	Non-US study	**>50%**^**a**^	Moderate
Davidson, 2015	Charleston, SC	6-mo RCT		38	48	**52.6% Black**, **47.4% Latinx**	57.90%	Moderate
Derose, 2019	South Los Angeles, CA	5-mo RCT	X	213	50.8	**45.3% Black**, **51.1% Latinx**	51.80%	Low-very low
Gonzalez-Sanchez, 2019	Spain	12-mo RCT		833	51.9	Non-US study	**70.20%**	High-moderate
Haufe, 2019	Wolfsburg, Germany	6-mo RCT		214	48.1	Non-US study	**73%**	Moderate
Kim, 2019	Seoul, South Korea	8-wk RCT		144	**77.7**	Non-US study	**82.26%**	Moderate-low
McManus, 2018	England	12-mo RCT		1182	**66.9**	Non-US study	**76.60%**	High
Newton, 2018	Baton Rouge, LA	6-mo cluster RCT		97	56	**100% Black**	**70.10%**	Very low
Or, 2016	Hong Kong	3-mo RCT	X	63	**69.5**	Non-US study	**88%**	Moderate
Skolarus, 2017	Flint, MI	6-mo RCT	X	94	58	**97% Black**	NR	Very low
Varleta, 2017	Santiago, Chile	6-mo RCT		314	60	Non-US study	**67.10%**	Low
Wakefield, 2011	Iowa City, IA	3-arm 6-mo RCT		302	**68**	1.99% Black, 0.99% AI/AN, 0.66% Latinx	46.70%	High
Zha, 2019	Newark, NJ	6-mo RCT	X	30	52.2	**96% Black**	NR	Moderate
Non-randomized clinical trials								
Brewer, 2019	Rochester and Minneapolis-St Paul, MN	10-wk single-group	X	50	49.6	**100% Black**	12.00%	Very low
Fukuoka, 2018	San Francisco, CA	8-wk pre-post		54	45.3	**100% Latinx**	31.50%	Very low
Jones, 2016	Lexington, KY	12-wk pre-post		40	58	**100% Black**	NR	Very low
Kim et al., 2019	Baltimore-Washington DC	6-mo single-group	X	247	60.9	**100% Korean American**	31.50%	Very low
Levin, 2019	Cleveland, OH	12-wk cohort	X	38	51.5	**73.7% Black**, 5.3% Hispanic, 2.6% Other	13.18 (2.69)b	Very low
Lewinski, 2019	Durham, NC	6-mo single-arm	X	141	56.9	**83.9% Black**, 3.4% Other	22.90%	Very low
Milani, 2017	New Orleans, LA	3-mo case-control		156	**68**	22.5% Black	NR	Very low
Orozco-Beltran, 2017	Spain	1-yr quasi-experimental		521	**70.4**	Non-US study	NR	Low
Patel, 2013	Washington, DC	10-mo cohort		50	53	**96% Black**	**79%**	Very low
Wenger, 2019	Atlanta, Georgia	6-mo pre-post	X	14	52	**100% Black**	NR	Very low

Bold text indicates the study met inclusion criteria for a vulnerable population.

*AI/AN* American Indian/Alaska Native, *GRADE* Grading of Recommendations, Assessment, Development and Evaluations, *HS educ.* high school education, *NR* not reported, *RCT* randomized control trial.

^a^Authors reported median (11.0) and IQR (9.0–13.0).

^b^Authors reported mean number of years of education.

Fifteen studies were randomized clinical trials (RCTs), five of which were pilot studies. There were 20 studies that met only one inclusion criterion: 12 met the race/ethnicity criterion, three met the age criterion, and five met the educational attainment criterion. Five studies met two out of the three criteria. Fifteen studies required participants to own technology (e.g., smartphone, cellular phone) and/or possess specific technology capabilities/abilities (e.g., text messaging, application use).

Fourteen studies used existing behavior theories or models to inform their intervention design (Supplementary Table 1). The most common models/theories used were the Social Cognitive Theory (4), the Transtheoretical Model (2), the Information-Motivation-Behavioral Skills Model (2), and the Self-Determination Theory (2).

Of the 25 studies, 17 studies measured blood pressure (BP) change only, two studies measured blood pressure control only, and six studies measured both blood pressure change and control. Only 11 studies used blood pressure as their primary outcome.

### Quality of included studies

Table 1 shows the results of the Grades of Recommendation, Assessment, Development, and Evaluation (GRADE) quality^63^ assessment of the 25 studies (detailed assessment in Supplementary Table 2). For the question of interest in this review, only two studies were rated as high quality. Several studies were considered lower quality for this review because of issues related to bias (not an RCT), indirectness (intervention included many components beyond mHealth), or imprecision (small sample size; wide confidence interval).

### Intervention features

The most common technology platform used was text messaging (15) followed by mobile applications (12). Table 2 describes studies’ intervention characteristics, categorized by the Practical Reviews In Self-Management Support (PRISMS) taxonomy^64^. A total of 20 studies included lifestyle advice and support. Fifteen studies provided information about hypertension self-management. Despite using mHealth, eleven studies still included an element of human coaching. Nine studies provided support with medication adherence (i.e., reminders); the same number of studies also included BP self-monitoring. Additionally, 15 studies provided training on the use of digital devices while 13 studies tailored their intervention based on patient’s knowledge or risk assessment.

**Table 2:** Intervention features according to Practical Reviews in Self-Management Support (PRISMS) taxonomy.

Study	Control	Tech platform	Intervention component							
			HTN educ.	BP monitoring	Med reminder	Equipment provision	Training on technology	Lifestyle advice	Other^a^	Human coaching
Randomized clinical trials										
Alonso-Domínguez, 2019	Diet & PA counseling	App				x		x		x
Buis, 2017	Usual care	SMS	x		x	x		x		
Chow, 2015	Community follow-up	SMS	x					x		
Davidson, 2015	Usual care	SMS		x	x	x		x		
Derose, 2019	Usual care	SMS						x	A2	
Gonzalez-Sanchez, 2019	Diet & PA counseling	App				x		x		
Haufe, 2019	Waiting list	App				x		x	A2, A8	x
Kim, 2019	Usual care	SMS	x					x		x (IG2 only)
McManus, 2018	Usual care	SMS		x		x	x			
Newton, 2018	Normal eating & exercise	SMS						x	A13	x
Or, 2016	Self-monitoring with log book	App	x	x		x	x	x		
Skolarus, 2017	AHA materials & SMS	SMS	x	x		x	x	x		
Varleta, 2017	No SMS	SMS	x		x			x		
Wakefield, 2011^b^	Usual care	App	x	x	x	x	x	x	A4	x
Zha, 2019	Usual care	App, Web		x		x	x			x
Non-randomized clinical trials										
Brewer, 2019	None	App	x			x			A13	
Fukuoka, 2018	None	App				x		x	A13	
Jones, 2016	None	SMS	x			x		x	A2, A8	
Kim et al., 2019	None	SMS		x		x	x	x	A9, A12	x
Lewinski, 2019	None	SMS	x		x			x	A4	x
Levin, 2019	None	SMS	x		x					
Milani, 2017	Usual care	SMS, App	x	x	x		x	x	A2, A3	x
Orozco-Beltran, 2017	None	App	x	x		x	x	x		x
Patel, 2013	None	App	x		x	x				x
Wenger, 2019	None	SMS, App	x	x	x	x		x		

*Abbreviations: SMS* (short message service), also known as text messaging.

^a^Refers to other PRISMS components: A2. Information about resources; A3. Provision of/agreement on specific action plans; A4. Regular clinic review; A8. Provision of easy access to advice/support; A9. Training/rehearsal to communicate with health care professionals; A12. Training/rehearsal for psych strategies; A13. Social support.

^b^Wakefield included a high-intensity (HIG) and low-intensity (LIG) intervention group. The HIG received daily health information tips and questions using a branching algorithm programmed into the device based on participant response. The LIG received up to two daily questions but no informational tips or questions based on the branching algorithm.

### BP outcome

Blood pressure outcomes (systolic blood pressure [SBP] change and BP control) appear in Table 3. All 15 RCTs reported SBP change; in these studies, the intervention group (IG) had greater SBP change than the control group (CG) in 7/15 studies when evaluating the higher intensity intervention groups (Table 3). Among the non-RCT studies, 4/8 reported significant SBP changes at the study conclusion. Two RCTs and six non-RCTs reported BP control; among these studies, both RCTs and 4/6 non-RCTs reported significant differences between groups.

**Table 3:** Blood Pressure Outcomes.

Study	Control or comparison group	Blood pressure outcome^a^	*p*-value
		SBP change and/or BP control	
Randomized clinical trials			
Alonso-Domínguez, 2019	Diet & PA counseling	At 12 mo, no difference in SBP change between CG and IG: −1.6 (−8.9, 5.6)	NS
Buis, 2017	Usual care	At 1 mo, no difference in mean SBP change: CG (−11.3) vs IG (−12.6)	0.78
Chow, 2015	Community follow-up	SBP change: At 6 mo, IG had greater SBP change than CG: −7.6 (−9.8, −5.4)	<0.001
		BP control: At 6 mo, IG had higher rates of BP control (79.2%) than CG (54.9%) with a 1.44 relative risk for control in IG	<0.001
Davidson, 2015	Usual care	SBP change: At 6 mo, IG had lower SBP than CG	<0.001
		BP control: At 6 mo, IG had higher SBP control rates than CG (94.4% vs 41.2%) and DBP control rates (94.4% vs 76.5%)	SBP: 0.003; DBP: 0.04
Derose, 2019	Usual care	At 6 mo, no difference in SBP change between CG and IG	NS
Gonzalez-Sanchez, 2019	Diet & PA counseling	At 12 mo, CG had greater SBP change than IG: −2.0 (−0.4, −3.6)	<0.05^b^
Haufe, 2019	Waiting list	At 6 mo, IG had greater SBP change than CG: −2.7 (−4.9, −0.4)	0.020
Kim, 2019	Usual care	At 8 wk, IG1 with text-messaging only had a similar SBP change vs CG. IG2 with text-messaging & coaching had greater SBP change than CG.	NS (IG1); <0.05 (IG2)
McManus, 2018	Usual care	At 12 mo, IG had greater SBP change than CG: −3.5 (−5.8, −1.2)	0.0029
Newton, 2018	Normal eating & exercise	At 6 mo, no difference in mean SBP change in CG (−0.4) vs IG (0.2)	0.90
Or, 2016	Self-monitoring with log book	At 3 mo, IG had a greater mean SBP change (−13.0) than CG (−5.4)	0.043
Skolarus, 2017	AHA materials & SMS	At 12 mo, no difference in SBP change between IG and CG: −3.1 (−14.4, 8.3)	0.60
Varleta, 2017	No SMS	At 6 mo, BP reduction higher in IG, but per authors inadequate power for statistical comparisons.	NS
Wakefield, 2011	Usual care	At 12 mo, the high-intensity intervention (HIG) but not low-intensity (LIG) had greater SBP change than CG.	HIG: 0.006 LIG: NS
Zha, 2019	Usual care	At 6 mo, no difference in mean SBP change between IG (−8.4) vs CG (−4.8)	NS
Non-randomized clinical trials			
Brewer, 2019	None	SBP change: At 28 wk, mean SBP (127.1) lower than baseline (133.3)	0.002
		BP control: At 28 wk, BP control (81.6%) higher than baseline (59.2%)	0.005
Fukuoka, 2018	None	At 2 mo, mean SBP (117.2) lower than baseline SBP (122.1)	<0.005
Jones, 2016	None	At 3 mo, mean SBP (138) lower than baseline SBP (147)	0.009
Kim et al., 2019	None	SBP change: At 6 mo, mean SBP (124.8) similar to baseline SBP (128.7)	NS
		BP control: At 6 mo, BP control (54.0%) improved vs baseline (42.8%)	0.021
Lewinski, 2019	None	At 6 mo, BP control (54.6%) similar to baseline (57.6%)	0.64
Levin, 2019	None	At 3 mo, mean SBP (136.0) similar to baseline SBP (133.0)	NS
Milani, 2017	Usual care	SBP change: At 90 d, mean SBP was lower in IG (133) than matched cohort (143) and compared to baseline SBP in IG (147)	<0.001
		BP control: At 90 d, 71% in IG achieved BP control compared to 31% of usual-care group	<0.001
Orozco-Beltran, 2017	None	At 1 yr, the rate of uncontrolled SBP (32.6% from 36.5%) and DBP (7.7% from 13.8%) improved	SBP: 0.001; DBP: 0.01
Patel, 2013	None	SBP change: At 6 mo, mean SBP (135) similar to pre-intervention (137)	NS
		BP control: At 6 mo, BP control (60%) similar to (66%) pre-intervention	NS
Wenger, 2019	None	At 6 mo, mean SBP (124) lower than baseline (131) but not powered for statistical analysis	NS

*Abbreviations: BP* (blood pressure); *CG* (control group); *DBP* (diastolic blood pressure); *IG* (intervention group); *NS* (non-significant); *SBP* (systolic blood pressure).

^a^Outcomes reported as described in each study. If no specific values are listed in the table, the studies did not provide specific values.

^b^Control group had better outcomes in this study.

When analyzing the high-intensity intervention groups in the RCTs, 1/6 studies that met inclusion criteria by race/ethnicity, 5/9 that met inclusion criteria by educational attainment, and 4/6 that met inclusion criteria by age had significant SBP decreases (Supplementary Fig. 1, left panel). Within the RCTs, 3/6 studies that used apps had a significant SBP decrease (when using the high-intensity intervention groups). Of the nine RCTs that used SMS, four had significant SBP decreases when evaluating the high-intensity intervention group (Supplementary Fig. 2, left panel).

**Fig. 2:**
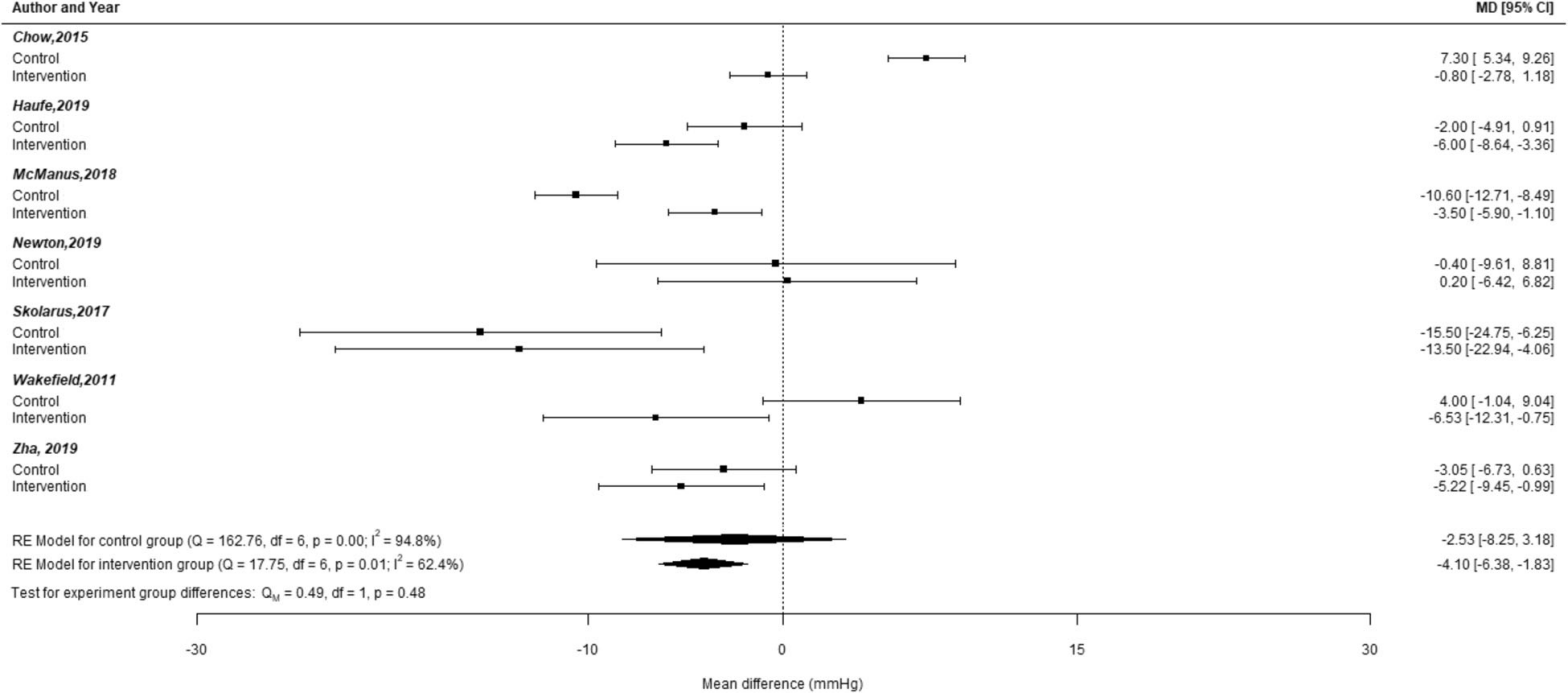
Plot of mean differences in SBP change between the intervention group and control group at 6-months for RCTs included in meta-analyses. Forest plot of the mean difference in systolic blood pressure (SBP) within the experimental group. The top portion of this figure shows the mean difference (MD) in SBP at 6 mo within the intervention or control group for each study. Error bars for each study signify the 95% confidence intervals for the mean difference within the control group or intervention group for each study. The control groups for all studies were combined to create an estimated average effect in a random-effects model. A similar procedure was done for the intervention groups. The summary polygons at the bottom of the plot show results of random-effects models. Among all participants in the intervention group, there was a statistically significant decrease in the mean difference for SBP change. However, this mean difference estimate was not significantly different from the estimate from the control group (test for experimental group difference: *p* = 0.48).

Meta-analyses of the seven RCTs that included a 6-month SBP change outcome showed that while the intervention groups had a significant decrease in SBP (mean difference = −4.10; 95% CI: −6.38, −1.38), this was not significantly different from the SBP change in control groups (*p* = 0.48, Fig. 2). The seven studies included in the meta-analyses used SMS (4) and mobile applications (3) and included populations that met inclusion criteria based on race/ethnicity (3), educational attainment (4), and age (2).

### Other outcomes: medication adherence, engagement, and satisfaction

Eleven studies reported medication adherence. Of these, 2/8 RCTs reported a significant impact on medication adherence while 2/3 non-RCTs reported a significant impact on medication adherence (Table 4). Only one of the two RCTs that had a significant impact on medication adherence also had a significant reduction in SBP; three of the six RCTs with no impact on medication adherence produced a significant reduction in SBP. The two non-RCTs with significant changes in medication adherence did not have a significant SBP change; the one non-RCT with a non-significant medication adherence change did have a significant SBP reduction.

**Table 4:** Medication adherence outcomes.

Study	Medication adherence measure	Medication adherence outcome	*p*-value
Randomized clinical trials			
Buis, 2017	Morisky Medication Adherence Scale (8-items)	At 1 mo, IG had higher mean medication adherence change vs CG: 0.9 vs 0.5	0.26
Davidson, 2015	Modified algorithm by Russell,	At 6 mo, medication adherence was 0.92 among IG	NR
Kim M, 2019	Morisky Medication Adherence Scale (4-items)	At 8 wk, IG1 with text-messaging only had similar medication adherence change vs CG. IG2 with text-messaging & coaching had greater medication adherence change than CG	IG1: NS IG2: <0.05
McManus, 2018	Medication Adherence Rating Scale	At 12 mo, no difference in medication adherence between IG and CG: 0.02 (−0.20, 0.25)	0.83
Skolarus, 2017	Morisky Medication Adherence Scale (1-item)	At 12 mo, no difference in medication adherence within IG	0.69
Varleta, 2017	Morisky-Green-Levine questionnaire (4-items)	At 6 mo, IG significantly improved adherence (49% to 62.3%) vs CG (59.3% to 51.4%)	IG: 0.01 CG: 0.10
Wakefield, 2011	Edwards Regimen Adherence Scale	At 12 mo, no difference in medication adherence among the high-intensity intervention (HIG), low-intensity (LIG), and CG	0.09
Zha, 2019	Medication Adherence Self-Efficacy Scale	At 6 mo, IG had higher mean medication adherence change vs CG: 69.17 vs 61.00	0.06
Non-randomized clinical trials			
Levin, 2019	Tablets Routine Questionnaire (TRQ), Electronic Cap (eCAP)	At 3 mo, mean anti-hypertensive nonadherence (21%) lower than baseline (43%) as reported by TRQ. eCAP did not show a significant difference	TRQ: <0.001 eCAP: NS
Milani, 2017	Questionnaire via electronic health record (MyChart)	At 3 mo, low medication adherence rate (16.5%) improved among IG from baseline (17%)	NR
Patel, 2013	Morisky Self-Reported Medication Scale (Morisky SMS) (4-items), Pharmacy Refill Rate (PRR)	At 6 mo, medication adherence (3.2) improved from baseline (2.4). PRR did not show a significant increase	Morisky SMS: 0.00 PRR: 0.06

Of the 25 studies included in this study, only 13 studies reported participant engagement and seven studies reported participant satisfaction. These studies reported variable levels of engagement (Table 5). Most studies support high initial rates of mHealth usage and deterioration of usage over time though most studies reported an average use of at least 50% at study end. Of these 13 studies, six reported a significant improvement in blood pressure outcomes at study end. In the studies that reported satisfaction, all reported 80%+ satisfaction on various measures of satisfaction, including ease of use, usefulness, and satisfaction (Table 6). Only one of these studies also reported a significant improvement in blood pressure.

**Table 5:** Engagement outcomes.

Study	Engagement
Chow, 2015	Read at least three-fourths of messages: 96%
Davidson, 2015	On-time BP adherence (% BP measured every 3 days): 86.2%
Fukuoka, 2018	Self-weighing and logging into the Fitbit app at least twice per week: 49.3%, self-weighing and logging into the Fitbit app at least once per week: 76.7%
Gonzalez-Sanchez, 2019	High app adherence (defined as >60 days): 56%, app used for less than a month: 28.2%
Haufe, 2019	% of IG logged a total amount of exercise ≥150 min/week: 48%
Levin, 2019	Mean % of valid responses^a^ to text-message prompts: 67%, mean % of valid responses^a^ to mood messages: 56%
Lewinski, 2019	Completed ≥4 phone calls: 98
Or, 2016	Uploaded measurements at least 3 days/week: 93% at 1-mo; 67% at 2-mo; 73% at 3-mo
Patel, 2013	Pill phone utilization (# of pills indicated as “taken” in a week/# of pills prescribed for that week): 63% at 1-wk; 54% at 12-weeks
Skolarus, 2017	Mean # of weeks participants responded to BP prompts (out of 26 weeks): 13.7, % who did not respond to any weeks of BP prompts: 17%, % who responded with their BP every week: 26%
Wakefield, 2011	# of days participants entered data via telehealth device: 125 days/182 days (69%) in the LIG; 127 days/182 days (70%) in the HIG
Wenger, 2019	Overall engagement: 1-mo: 85–100%, 6-mo: 50–78%
Zha, 2019	BP monitoring adherence rate at home: 71% in IG; 65% in CG

*Abbreviations: BP* (blood pressure); *HIG* (high-intensity group); *IG* (intervention group); *LIG* (low-intensity group).

^a^Valid responses: the system had protections against multiple or inaccurate responses.

**Table 6:** Satisfaction and acceptance outcomes.

Study	Satisfaction or acceptance outcome
Buis, 2017	Satisfied: 94%
	Easy to use: 98%
	Recommend: 94%
	Would continue using text message reminders: 85%
Chow, 2015	Easy to use: 97%
	Useful: 91%
Levin, 2019	Useful: 87%
	Recommend: 100%
	Would continue using: 95%
Newton, 2018	Satisfied: The average score for SMS text messages was 1.4 (0.67) (lower scores indicating high levels of satisfaction)
	% of participants who requested SMS text messages be stopped: 6.1%
Patel, 2013	Satisfied: Median score was 4.6 out of 5.0 (high levels of satisfaction)
Skolarus, 2017	Satisfied: 100%
	Easy to use: 84%
	Would continue receiving text messages: 84%
	Consistent with the language used: 90%
	Helpful tips to manage BP: 95%
Wenger, 2019	Easy to use: 91%
	Useful: 92%
	Appropriate in frequency: 92%

## Discussion

Despite the explosion of interest in mHealth, the potential for innovations to address gaps in blood pressure control, and no study design restrictions in our search criteria, we were able to identify only 25 studies that included older, lower educational attainment, and non-White populations—key populations that are more susceptible to poor blood pressure control. Unfortunately, this is consistent with decades of research showing that low-income and minority populations are less likely to be included in clinical studies^65^. Despite growing recognition of the importance of trial diversity and required age and racial/ethnicity diversity in National Institutes of Health-funded studies, the continued poor representation of diverse populations suggests that these efforts have been insufficient to increase trial diversity for digital health trials, particularly for high-quality study designs. In this review, only 13 studies met inclusion criteria based on race/ethnicity, and most of these studies utilized either non-RCT designs or were small pilot RCTs, thereby limiting the generalizability of these findings. Moreover, it is important to note that this paucity of studies limits our understanding of the importance of digital literacy and access, given that these same populations are known to experience disparities in digital access, such as lower rates of broadband access and/or mobile device ownership in the United States^17^.

The 15 studies in this review that reported engagement or satisfaction outcomes show overwhelming interest and satisfaction with digital/mobile interventions—a finding that is consistent with several studies demonstrating that many older, lower socioeconomic status, and minority patients are interested in digital health tools^66,67^. Therefore, despite evidence that suggests these populations may have a lower preference for the digital health tools and digital communication strategies that are currently available^9,67–69^, there is still significant interest. We cannot ignore this interest and instead must ensure that digital health tools are accessible and usable for these diverse populations. In addition, the included studies in this review serve as clear examples of how to provide straightforward support to participants in accessing the digital intervention successfully. It is imperative that future digital health studies include and focus on populations that experience disparities in health outcomes so we can ensure that interventions are designed for use in all populations and that they do not exacerbate disparities^70,71^.

Many studies failed to meet our inclusion criteria because they did not report the educational attainment and/or race/ethnicity of their study population. Even after attempts to contact 50+ authors to include additional studies, over half of the authors reported that they had not collected this additional information. These sociodemographic factors have been consistently shown to be correlated with health outcomes; it is crucial that researchers more reliably report these data to understand the external validity of any study, particularly among populations with a higher prevalence of hypertension. Similarly, few studies reported information related to literacy, particularly digital literacy^72^, a crucial factor known to impact the effectiveness of digital health interventions^11,73^. These factors should be increasingly recognized as core to describing any study’s participants to better understand generalizability for diverse populations.

Some of the 25 studies included in this review showed success in improving blood pressure, particularly among older populations or those with limited educational attainment, but the variability in study design, intervention, primary outcome, and timing of outcome ascertainment made it difficult to conduct meta-analyses. We were therefore only able to include seven studies in our meta-regression analyses, and even within these seven studies, there was high variability in the study population and intervention design. This type of variability will likely continue to limit future efforts to determine a summary estimate (e.g., average) or examine the overall effectiveness of mHealth interventions in chronic disease management.

An additional challenge to summarizing the literature was the high variability in study outcomes. Some studies focused their primary outcome on blood pressure control while others focused on blood pressure change. The timing of the outcome also was not consistent and included 1-month, 3-months, 6-months, and 1-year time points. Lack of outcome uniformity is an ongoing challenge in many meta-analyses and reviews^74^. Similarly, few studies if any reported long-term outcomes data. While there are models that can determine long-term clinical outcomes (e.g., mortality, cardiovascular morbidity, heart failure) based on blood pressure control, these studies could be strengthened by direct measurement of these clinical outcomes.

Many of the interventions assessed in these studies had multiple components. While multi-modal interventions are more effective at encouraging the type of behavior change pursued in these studies, it poses challenges to determine which aspects of the intervention are most impactful. One way to address this is by using behavior change frameworks and theories that facilitate a more systematic way of reporting interventions so that researchers and clinicians can compare the various aspects of each intervention. Despite the growth of implementation science and behavior change frameworks^75^, nearly half of the studies did not use any framework in the design of their intervention. Future researchers need to use an implementation science lens, particularly given the variation in intervention design, to facilitate understanding and consensus on which types of interventions and intervention components are most important for different groups of patients.

This review is limited by an approach that relied primarily on single-reviewer screening of title/abstract for inclusion, failure to include some relevant databases (e.g., CINAHL, Scopus), and inability to acquire full text for several articles which may have resulted in a biased inclusion of studies and early exclusion of studies although we encouraged reviewers to be conservative when excluding studies in the early stages. We also included studies with variation in study design, study population, intervention, or measured outcome, but despite these variations, we believe the relatively inclusive nature of our article selection provides a better assessment of the state of mHealth research for blood pressure self-management in populations that are older, non-White, or have limited educational attainment. We likely also missed relevant studies since many did not report the race/ethnicity or educational attainment of their participants; though we attempted to contact authors, we did not receive a response from all the authors.

## Conclusions

Despite the limitations of this review, we believe this paper adds to the digital health literature by focusing specifically on the effectiveness of mobile health strategies for self-management of hypertension in vulnerable populations that experience outcome disparities (older, minority, and limited educational attainment patients). Hypertension is a highly prevalent chronic disease, and blood pressure control is often worse in these same populations that are underrepresented in digital health studies. While we found these interventions to show some promise, particularly among older populations or those with limited educational attainment, this review reinforces the need to specifically include diverse populations in high-quality clinical trials on digital health interventions. The continued lack of racial/ethnic diversity in large-high-quality clinical trials is indefensible, particularly since the studies in this review clearly show high participant interest, engagement, and satisfaction in these populations. Given the variability in intervention design, it is also crucial that mHealth researchers begin to employ implementation science frameworks to facilitate comparison between different multi-modal studies. There are likely many mHealth studies being designed or in progress. We hope that researchers leading these trials will learn from prior studies by recruiting a more diverse cohort; collecting the necessary additional sociodemographic traits (e.g., education, digital literacy, race/ethnicity) to understand generalizability; and employing behavior change or implementation science frameworks to better understand which intervention components are most impactful for which individuals.

## Methods

This was a systematic review and meta-analyses conducted in collaboration with a clinical librarian and registered in advance on PROSPERO under study number CRD42017055836. We followed the Preferred Reporting Items for Systematic Reviews and Meta-Analyses (PRISMA) guidelines for conducting and reporting items for systematic reviews (Supplementary Data 1 and 2).

### Search strategy

We created a search strategy using keywords and controlled vocabulary, including MeSH and Emtree terms, for each concept of our research question, namely mHealth, and hypertension. We applied Boolean logic by combining similar terms with OR and using AND between each concept; for example, (“Mobile Applications” [Mesh] OR mhealth) AND (“Hypertension” [Mesh] OR “blood pressure”). Complete search strategies for each database can be found in Supplementary Data 3. Our original search also included a concept for vulnerable populations; however, the inclusion of this concept resulted in the elimination of several key studies from our results. As a result, we removed this search concept and manually excluded studies that did not focus on vulnerable populations during our screening process. Because many of our search terms represent new technologies that may exist as MeSH subheadings or be expressed differently by authors and MEDLINE indexers, we used a text word [tw] search in PubMed to ensure greater search sensitivity. Our search strategy was developed using an iterative process whereby the research team examined results for each search term and eliminated terms that produced irrelevant results. We developed the search strategy for PubMed and translated it to other databases, and the final search strategy was peer-reviewed by a second librarian using the Peer Review of Electronic Search Strategies (PRESS) guidelines.

We conducted a systematic search for studies involving mHealth and hypertension from 2005 to present in PubMed, Embase, Web of Science, and Google Scholar on August 9, 2017, and updated on July 23, 2019. We limited the search from 2005 to 2019 to reflect the fact that mobile phones were not widely used before 2005. No language limits were used. We searched for gray literature, including conference proceedings, websites, and government reports, using Google and organizational websites like the Agency for Healthcare Research and Quality and the California Healthcare Foundation. Hand searching of reference lists and key journals was also completed.

### Eligibility criteria

#### Inclusion criteria

Our review focuses on studies in urban populations in high-income countries, as defined by the World Bank because barriers to care and the practice of medicine in rural areas and low-income countries are different. Within low and middle-income countries, the context of and infrastructure for digital care is notably different from high-income countries^76^. In rural areas, which tend to have inadequate access to and supply of healthcare clinicians, mHealth interventions often focus on the replacement of in-person clinical care rather than supplementing clinical care with self-management, which is the focus of this review^77^. We defined “urban” according to the U.S. Census Bureau’s definition for US locations only. Included studies had to focus on at least one population with known disparities in use of digital health tools, defined by any one of the following three characteristics: age (mean age >65 years); education (>60% high school education or less); and/or race/ethnicity (<50% non-Hispanic White for US studies). In addition, studies were included if their self-management interventions involved active engagement with mobile technology such as a phone application, text messaging (short messaging service, or SMS), or a web-based platform. All studies were required to include blood pressure as an outcome.

#### Exclusion criteria

We excluded studies conducted in rural settings and non-English studies. For US locations, rural was defined by the US Census Bureau. Non-US locations were excluded if they self-identified as rural. We excluded studies that focused on pulmonary and gestational hypertension. In addition, studies were excluded if evaluations did not include clinical outcomes (e.g., if evaluation focused exclusively on app usability or user satisfaction) or if no self-management intervention was described. We also excluded studies that did not measure blood pressure pre-and/or post-intervention. Studies without primary data (e.g., protocols, reviews) and studies without full text (e.g., unable to acquire full text and conference abstracts only) were excluded.

### Study selection

Three reviewers conducted the initial screening based on title and abstract to determine if studies met inclusion for full-text review. All three reviewers reviewed ~5% of the articles found and reached a moderate agreement (Cohen’s kappa = 0.475) before independently screening the remaining articles based on title and abstract. Afterward, reviewers double-screened the remaining studies in full-text form and assessed against the inclusion and exclusion criteria. All studies included in this report were double-screened and disagreements were reconciled through discussion until a consensus was made. During the full-text screen, we contacted 57 study authors to request more information about their study population and/or intervention to determine inclusion; we contacted authors three times and received responses from 28 authors. Two reviewers determined final inclusion for analysis.

### Data extraction

Two reviewers double-extracted the data independently. Any discrepancies were discussed and reconciled. We created a standardized form to extract the following data: study characteristics (authors, year of publication, study location, study design and follow-up period, intervention design process); participant information; details about the intervention primarily characterized using the Practical Reviews In Self-Management Support (PRISMS) taxonomy, which describes self-management support interventions in 14 components;^64^ and blood pressure, medication adherence, engagement, and satisfaction outcomes. We supplemented the PRISMS taxonomy with additional intervention components: training on the use of technology and human coaching based on our prior studies showing the importance of these strategies in lower-income or limited health literate populations^78,79^. The PRISMS taxonomy was assessed specifically in regard to hypertension self-management. For example, a study would be marked as using self-management component A5 (monitoring of condition with feedback) only if it involved blood pressure monitoring. A study that asked participants to monitor glucose, weight, or physical activity would not be marked as using this self-management strategy.

### Assessment of risk of bias and quality

Each study was evaluated using the Grades of Recommendation, Assessment, Development, and Evaluation (GRADE) methodology system for assessing methodological quality^63^. The GRADE system assesses the quality of evidence as either high, moderate, low, or very low. This quality assessment is based on the specific review question; therefore, a high-quality study may not be assessed as high quality for a specific question if not designed to directly answer that review question. In brief, randomized controlled trials start as high quality and observational studies start as low quality. Studies may decrease in quality of evidence after considering the risk of bias, indirectness, and imprecision. Indirectness includes considerations such as whether the intervention applies to the question of interest; for this study, if the intervention included substantial non-mHealth-related components, we assessed that the intervention suffered from indirectness since it is possible the non-mHealth components of the intervention drove the outcome change. Studies may increase in quality of evidence based on strong evidence of association, evidence of a dose-response gradient, and confounders minimizing the effect. Two authors independently assessed for risk of bias and the quality of the evidence for study using a standardized checklist^80^. Any disagreements were resolved by discussion.

### Outcomes

The primary outcomes changed in systolic blood pressure (SBP) or blood pressure (BP) control (as defined by each study). Secondary outcome measures include medication adherence, satisfaction, and engagement.

### Meta-analyses

The results from each study were described. The seven randomized clinical trials that reported SBP change at six months were analyzed in two random-effects meta-analyses models, one model for the intervention group and the model for the control group (Detailed data in Supplementary Table 3). We fitted a meta-regression model to test for the difference in reported SBP changes to determine the effect of the intervention. All analyses were done using the package metafor from R (version 2.4–0).

### Reporting summary

Further information on research design is available in the Nature Research Reporting Summary linked to this article.

## Supplementary information

Supplementary Information

Reporting Summary

## Data Availability

The data supporting the findings of this study are available within the paper and its Supplementary Information files.

## References

[1] WHO Global Observatory for eHealth & World Health Organization. *mHealth: New Horizons for Health Through Mobile Technologies* (World Health Organization, 2011).

[2] Steinhubl SR, Muse ED, Topol EJ (2013). Can mobile health technologies transform health care?. J. Am. Med. Assoc..

[3] de Jongh T, Gurol-Urganci I, Vodopivec-Jamsek V, Car J, Atun R (2012). Mobile phone messaging for facilitating self-management of long-term illnesses. Cochrane Database Syst. Rev..

[4] Bonoto BC (2017). Efficacy of mobile apps to support the care of patients with diabetes mellitus: a systematic review and meta-analysis of randomized controlled trials. JMIR MHealth UHealth.

[5] Widmer RJ (2015). Digital health interventions for the prevention of cardiovascular disease: a systematic review and meta-analysis. Mayo Clin. Proc..

[6] Mahmood A, Kedia S, Wyant DK, Ahn S, Bhuyan SS (2019). Use of mobile health applications for health-promoting behavior among individuals with chronic medical conditions. Digit. Health.

[7] Langford, A. T. et al. Mobile phone ownership, health apps, and tablet use in US adults with a self-reported history of hypertension: cross-sectional study. *JMIR Mhealth Uhealth* **7**, e12228 (2019). 10.2196/12228 PMC6682274 31344667

[8] Langford A, Orellana K, Kalinowski J, Aird C, Buderer N (2020). Use of tablets and smartphones to support medical decision making in us adults: cross-sectional study. JMIR MHealth UHealth.

[9] Khoong EC, Rivadeneira NA, Hiatt RA, Sarkar U (2020). The use of technology for communicating with clinicians or seeking health information in a multilingual urban cohort: cross-sectional survey. J. Med. Internet Res..

[10] Nouri SS (2020). Patient characteristics associated with objective measures of digital health tool use in the US: a literature review. J. Am. Med. Inform. Assoc..

[11] Sarkar U (2016). Usability of commercially-available mobile applications for diverse patients. J. Gen. Intern. Med..

[12] Tieu L (2017). Online patient websites for electronic health record access among vulnerable populations: portals to nowhere?. J. Am. Med. Inform. Assoc..

[13] Martin T (2012). Assessing mHealth: opportunities and barriers to patient engagement. J. Health Care Poor Underserved.

[14] Marcolino MS (2018). The impact of mhealth interventions: systematic review of systematic reviews. JMIR Mhealth Uhealth.

[15] Piette JD (2015). Mobile health devices as tools for worldwide cardiovascular risk reduction and disease management. Circulation.

[16] Liu Patrick (2020). Use of mobile health applications in low-income populations. Circ. Cardiovasc. Qual. Outcomes.

[17] Mobile fact sheet. *Pew Research Center* https://www.pewresearch.org/internet/fact-sheet/mobile/ (2019).

[18] Ramirez V (2016). Assessing the use of mobile health technology by patients: an observational study in primary care clinics. JMIR MHealth UHealth.

[19] Moore SL (2014). A mobile health infrastructure to support underserved patients with chronic disease. Healthc. Amst. Neth..

[20] Silva BMC, Rodrigues JJPC, de la Torre Díez I, López-Coronado M, Saleem K (2015). Mobile-health: a review of current state in 2015. J. Biomed. Inform..

[21] Muntner P (2020). Trends in blood pressure control among US adults with hypertension, 1999-2000 to 2017-2018. JAMA.

[22] Valderrama A, Gillespie C, Mercado C (2013). Racial/ethnic disparities in the awareness, treatment, and control of hypertension—United States, 2003–2010. Morb. Mortal. Wkly. Rep..

[23] Wang Z (2014). Relation of socioeconomic status to hypertension occurrence. Int. J. Cardiol..

[24] Liu X, Rodriguez CJ, Wang K (2015). Prevalence and trends of isolated systolic hypertension among untreated adults in the United States. J. Am. Soc. Hypertens..

[25] Fang J, Yang Q, Ayala C, Loustalot F (2014). Disparities in access to care among US adults with self-reported hypertension. Am. J. Hypertens..

[26] Khatib R (2014). Patient and healthcare provider barriers to hypertension awareness, treatment and follow up: a systematic review and meta-analysis of qualitative and quantitative studies. PLoS ONE.

[27] Omboni S, Ferrari R (2015). The role of telemedicine in hypertension management: focus on blood pressure telemonitoring. Curr. Hypertens. Rep..

[28] Sivakumaran D, Earle KA (2014). Telemonitoring: use in the management of hypertension. Vasc. Health Risk Manag..

[29] Posadzki P (2016). Automated telephone communication systems for preventive healthcare and management of long-term conditions. Cochrane Database Syst. Rev..

[30] Tucker KL (2017). Self-monitoring of blood pressure in hypertension: a systematic review and individual patient data meta-analysis. PLOS Med..

[31] Alessa T, Abdi S, Hawley MS, de Witte L (2018). Mobile Apps to Support the Self-Management of Hypertension: Systematic Review of Effectiveness, Usability, and User Satisfaction. JMIR MHealth UHealth.

[32] Li R, Liang N, Bu F, Hesketh T (2020). The effectiveness of self-management of hypertension in adults using mobile health: systematic review and meta-analysis. JMIR MHealth UHealth.

[33] Pratap A (2020). Indicators of retention in remote digital health studies: a cross-study evaluation of 100,000 participants. Npj Digit. Med..

[34] Alonso-Dominguez, R. et al. Effectiveness of a multifactorial intervention in increasing adherence to the Mediterranean diet among patients with diabetes mellitus type 2: a controlled and randomized study (EMID study). *Nutrients* **11**, 162 (2019). 10.3390/nu11010162 PMC6357113 30646500

[35] Alonso-Domínguez R (2019). Effect of a multifactorial intervention on the increase in physical activity in subjects with type 2 diabetes mellitus: a randomized clinical trial (EMID study). Eur. J. Cardiovasc. Nurs..

[36] Alonso-Dominguez R (2019). Acute effect of healthy walking on arterial stiffness in patients with type 2 diabetes and differences by age and sex: a pre-post intervention study. BMC Cardiovasc. Disord..

[37] Brewer, L. C. et al. Improving cardiovascular health among African-Americans through a community-based mobile health lifestyle intervention: the faith! (fostering African-American improvement in total health) app! Pilot study. *Circulation* **34**, 1376–1378 (2018).

[38] Buis LR (2019). Improving blood pressure among African Americans with hypertension using a mobile health approach (the mi-bp app): protocol for a randomized controlled trial. JMIR Res. Protoc..

[39] Chow CK (2015). Effect of lifestyle-focused text messaging on risk factor modification in patients with coronary heart disease: a randomized clinical trial. JAMA.

[40] Davidson TM (2015). Evaluation of an mhealth medication regimen self-management program for African American and Hispanic uncontrolled hypertensives. J. Pers. Med..

[41] Derose KP (2019). Eat, pray, move: a pilot cluster randomized controlled trial of a multilevel church-based intervention to address obesity among African Americans and Latinos. Am. J. Health Promot..

[42] Fukuoka Y, Vittinghoff E, Hooper J (2018). A weight loss intervention using a commercial mobile application in Latino Americans-adelgaza trial. Transl. Behav. Med..

[43] Gonzalez-Sanchez J (2019). Using a smartphone app in changing cardiovascular risk factors: A randomized controlled trial (EVIDENT II study). Int J. Med. Inf..

[44] Haufe S (2019). Telemonitoring-supported exercise training, metabolic syndrome severity, and work ability in company employees: a randomised controlled trial. Lancet Public Health.

[45] Jones AR, Moser DK, Hatcher J (2018). Using text messages to promote health in African-Americans: #HeartHealthyandCancerFree. Ethn. Health.

[46] Kim M (2019). Effects of customized long-message service and phone-based health-coaching on elderly people with hypertension. Iran. J. Public Health.

[47] Kim MT (2019). Motivating people to sustain healthy lifestyles using persuasive technology: a pilot study of Korean Americans with prediabetes and type 2 diabetes. Patient Educ. Couns..

[48] Levin JB (2019). Outcomes of psychoeducation and a text messaging adherence intervention among individuals with hypertension and bipolar disorder. Psychiatr. Serv..

[49] Lewinski AA (2019). Addressing diabetes and poorly controlled hypertension: pragmatic mhealth self-management intervention. J. Med Internet Res..

[50] McManus RJ (2018). Efficacy of self-monitored blood pressure, with or without telemonitoring, for titration of antihypertensive medication (TASMINH4): an unmasked randomised controlled trial. Lancet.

[51] Milani RV, Lavie CJ, Bober RM, Milani AR, Ventura HO (2017). Improving hypertension control and patient engagement using digital tools. Am. J. Med..

[52] Newton RL (2018). A church-based weight loss intervention in African American adults using text messages (LEAN study): cluster randomized controlled trial. J. Med Internet Res.

[53] Or C, Tao D (2016). A 3-month randomized controlled pilot trial of a patient-centered, computer-based self-monitoring system for the care of type 2 diabetes mellitus and hypertension. J. Med. Syst..

[54] Orozco-Beltran D, Sanchez-Molla M, Sanchez JJ, Mira JJ (2017). Telemedicine in primary care for patients with chronic conditions: the valcronic quasi-experimental study. J. Med. Internet Res.

[55] Patel S (2013). Mobilizing your medications: an automated medication reminder application for mobile phones and hypertension medication adherence in a high-risk urban population. J. Diabetes Sci. Technol..

[56] Recio-Rodríguez JI (2019). Combined use of a healthy lifestyle smartphone application and usual primary care counseling to improve arterial stiffness, blood pressure and wave reflections: a randomized controlled trial (EVIDENT II study). Hypertens. Res..

[57] Skolarus LE (2018). Reach out churches: a community-based participatory research pilot trial to assess the feasibility of a mobile health technology intervention to reduce blood pressure among African Americans. Health Promot Pr..

[58] Varleta P (2017). Mobile phone text messaging improves antihypertensive drug adherence in the community. J. Clin. Hypertens..

[59] Wakefield BJ (2011). Effectiveness of home telehealth in comorbid diabetes and hypertension: a randomized, controlled trial. Telemed. J. E-Health . J. Am. Telemed. Assoc..

[60] Wakefield BJ (2012). Outcomes of a home telehealth intervention for patients with diabetes and hypertension. Telemed. E-Health.

[61] Wenger NK, Williams OO, Parashar S (2019). SMARTWOMAN: feasibility assessment of a smartphone app to control cardiovascular risk factors in vulnerable diabetic women. Clin. Cardiol..

[62] Zha, P. *et al*. Utilizing a mobile health intervention to manage hypertension in an underserved community. *West J. Nurs. Res*. 193945919847937, 10.1177/0193945919847937 (2019). 10.1177/0193945919847937 31057081

[63] Guyatt GH (2008). GRADE: an emerging consensus on rating quality of evidence and strength of recommendations. BMJ.

[64] Pearce, G. et al. The PRISMS taxonomy of self-management support: derivation of a novel taxonomy and initial testing of its utility. *J. Health Serv. Res. Policy*10.1177/1355819615602725 (2015). 10.1177/1355819615602725 26377727

[65] Clark LT (2019). Increasing diversity in clinical trials: overcoming critical barriers. Curr. Probl. Cardiol..

[66] Khoong, E. C. et al. Patient interest in and barriers to telemedicine video visits in a multilingual urban safety-net system. *J. Am. Med. Inform. Assoc*. ocaa234, 10.1093/jamia/ocaa234 (2020). 10.1093/jamia/ocaa234 PMC7883976 33164063

[67] Khoong EC (2019). Health information-seeking behaviors and preferences of a diverse, multilingual urban cohort. Med. Care.

[68] Gordon NP, Crouch E (2019). Digital information technology use and patient preferences for internet-based health education modalities: cross-sectional survey study of middle-aged and older adults with chronic health conditions. JMIR Aging.

[69] Knowles B, Hanson VL (2018). The wisdom of older technology (non)users. Commun. ACM.

[70] Lyles C, Schillinger D, Sarkar U (2015). Connecting the dots: health information technology expansion and health disparities. PLoS Med..

[71] Borg K, Boulet M, Smith L, Bragge P (2019). Digital inclusion & health communication: a rapid review of literature. Health Commun..

[72] Harris K, Jacobs G, Reeder J (2019). Health systems and adult basic education: a critical partnership in supporting digital health literacy. Health Lit. Res. Pract..

[73] Sieck CJ (2021). Digital inclusion as a social determinant of health. Npj Digit. Med..

[74] Saldanha IJ (2020). Outcome choice and definition in systematic reviews leads to few eligible studies included in meta-analyses: a case study. BMC Med. Res. Methodol..

[75] Tabak RG, Khoong EC, Chambers DA, Brownson RC (2012). Bridging research and practice: models for dissemination and implementation research. Am. J. Prev. Med..

[76] Mills A (2014). Health care systems in low- and middle-income countries. N. Engl. J. Med..

[77] Douthit N, Kiv S, Dwolatzky T, Biswas S (2015). Exposing some important barriers to health care access in the rural USA. Public Health.

[78] Fields J (2021). In-home technology training among socially isolated older adults: findings from the tech allies program. J. Appl. Gerontol..

[79] Lyles CR (2019). A randomized trial to train vulnerable primary care patients to use a patient portal. J. Am. Board Fam. Med..

[80] Meader N (2014). A checklist designed to aid consistency and reproducibility of GRADE assessments: development and pilot validation. Syst. Rev..

