# Supplementary material for: Mobile health strategies for blood pressure self-management in urban populations with digital barriers: systematic review and meta-analyses
Source: NPJ Digit Med. 2021 Jul 22;4:114. doi: 10.1038/s41746-021-00486-5 (PMC8298448; doi:10.1038/s41746-021-00486-5)
Supplement: Supplementary file 1 — Supplementary Information [file 41746_2021_486_MOESM1_ESM.pdf]

Contained within:

<u>Supplementary Information</u>

Supplementary Data 1: PRISMA checklist
Supplementary Data 2: PRISMA-S checklist
Supplementary Data 3: Search strategy details

Supplementary Table 1: Studies that used theories or models to inform intervention design
Supplementary Table 2: GRADE Assessment
Supplementary Table 3: Data included in meta-regression analysis

Supplementary Figure 1: Difference in mean systolic blood pressure between intervention and control group by population in (a) RCT studies or between the intervention group and the comparator group in (b) non-RCT studies
Supplementary Figure 2: Difference in mean systolic blood pressure between intervention and control group by technology platform in (a) RCT studies or between the intervention group and the comparator group in (b) non-RCT studies

Supplementary Data 1: Preferred Reporting Items for Systematic reviews and Meta-Analyses (PRISMA) Checklist

Section and Topic	Item #	Checklist item	Location where item is reported
**TITLE**			
Title	1	Identify the report as a systematic review.	1
**ABSTRACT**			
Abstract	2	See the PRISMA 2020 for Abstracts checklist.	3
**INTRODUCTION**			
Rationale	3	Describe the rationale for the review in the context of existing knowledge.	4
Objectives	4	Provide an explicit statement of the objective(s) or question(s) the review addresses.	4
**METHODS**			
Eligibility criteria	5	Specify the inclusion and exclusion criteria for the review and how studies were grouped for the syntheses.	10
Information sources	6	Specify all databases, registers, websites, organisations, reference lists and other sources searched or consulted to identify studies. Specify the date when each source was last searched or consulted.	9
Search strategy	7	Present the full search strategies for all databases, registers and websites, including any filters and limits used.	Appendix Text 3
Selection process	8	Specify the methods used to decide whether a study met the inclusion criteria of the review, including how many reviewers screened each record and each report retrieved, whether they worked independently, and if applicable, details of automation tools used in the process.	10
Data collection process	9	Specify the methods used to collect data from reports, including how many reviewers collected data from each report, whether they worked independently, any processes for obtaining or confirming data from study investigators, and if applicable, details of automation tools used in the process.	10-11
Data items	10a	List and define all outcomes for which data were sought. Specify whether all results that were compatible with each outcome domain in each study were sought (e.g. for all measures, time points, analyses), and if not, the methods used to decide which results to collect.	10-11
	10b	List and define all other variables for which data were sought (e.g. participant and intervention characteristics, funding sources). Describe any assumptions made about any missing or unclear information.	10-11
Study risk of bias assessment	11	Specify the methods used to assess risk of bias in the included studies, including details of the tool(s) used, how many reviewers assessed each study and whether they worked independently, and if applicable, details of automation tools used in the process.	11
Effect measures	12	Specify for each outcome the effect measure(s) (e.g. risk ratio, mean difference) used in the synthesis or presentation of results.	11-12
Synthesis methods	13a	Describe the processes used to decide which studies were eligible for each synthesis (e.g. tabulating the study intervention characteristics and comparing against the planned groups for each synthesis (item #5)).	10-11
	13b	Describe any methods required to prepare the data for presentation or synthesis, such as handling of missing summary statistics, or data conversions.	n/a
	13c	Describe any methods used to tabulate or visually display results of individual studies and syntheses.	n/a
	13d	Describe any methods used to synthesize results and provide a rationale for the choice(s). If meta-analysis was performed, describe the model(s), method(s) to identify the presence and extent of statistical heterogeneity, and software package(s) used.	11-12
	13e	Describe any methods used to explore possible causes of heterogeneity among study results (e.g. subgroup analysis, meta-regression).	n/a
	13f	Describe any sensitivity analyses conducted to assess robustness of the synthesized results.	n/a
Reporting bias assessment	14	Describe any methods used to assess risk of bias due to missing results in a synthesis (arising from reporting biases).	n/a
Certainty assessment	15	Describe any methods used to assess certainty (or confidence) in the body of evidence for an outcome.	11
**RESULTS**			

Section and Topic	Item #	Checklist item	Location where item is reported
Study selection	16a	Describe the results of the search and selection process, from the number of records identified in the search to the number of studies included in the review, ideally using a flow diagram.	Figure 1
	16b	Cite studies that might appear to meet the inclusion criteria, but which were excluded, and explain why they were excluded.	n/a
Study characteristics	17	Cite each included study and present its characteristics.	Tables 1-6
Risk of bias in studies	18	Present assessments of risk of bias for each included study.	Appendix Table 2
Results of individual studies	19	For all outcomes, present, for each study: (a) summary statistics for each group (where appropriate) and (b) an effect estimate and its precision (e.g. confidence/credible interval), ideally using structured tables or plots.	Tables 1-6, Figure 2
Results of syntheses	20a	For each synthesis, briefly summarise the characteristics and risk of bias among contributing studies.	p 5, Appendix Table 2
	20b	Present results of all statistical syntheses conducted. If meta-analysis was done, present for each the summary estimate and its precision (e.g. confidence/credible interval) and measures of statistical heterogeneity. If comparing groups, describe the direction of the effect.	p 6, Figure 2
	20c	Present results of all investigations of possible causes of heterogeneity among study results.	n/a
	20d	Present results of all sensitivity analyses conducted to assess the robustness of the synthesized results.	n/a
Reporting biases	21	Present assessments of risk of bias due to missing results (arising from reporting biases) for each synthesis assessed.	n/a
Certainty of evidence	22	Present assessments of certainty (or confidence) in the body of evidence for each outcome assessed.	n/a
**DISCUSSION**			
Discussion	23a	Provide a general interpretation of the results in the context of other evidence.	7-8
	23b	Discuss any limitations of the evidence included in the review.	8
	23c	Discuss any limitations of the review processes used.	8-9
	23d	Discuss implications of the results for practice, policy, and future research.	11-12
**OTHER INFORMATION**			
Registration and protocol	24a	Provide registration information for the review, including register name and registration number, or state that the review was not registered.	9
	24b	Indicate where the review protocol can be accessed, or state that a protocol was not prepared.	9
	24c	Describe and explain any amendments to information provided at registration or in the protocol.	n/a
Support	25	Describe sources of financial or non-financial support for the review, and the role of the funders or sponsors in the review.	13
Competing interests	26	Declare any competing interests of review authors.	13
Availability of data, code and other materials	27	Report which of the following are publicly available and where they can be found: template data collection forms; data extracted from included studies; data used for all analyses; analytic code; any other materials used in the review.	Appendix Table 3

From: Page MJ, McKenzie JE, Bossuyt PM, Boutron I, Hoffmann TC, Mulrow CD, et al. The PRISMA 2020 statement: an updated guideline for reporting systematic reviews. BMJ 2021;372:n71. doi: 10.1136/bmj.n71

Supplementary Data 2: PRISMA-S checklist

Section/topic	#	Checklist item	Location(s) Reported
**INFORMATION SOURCES AND METHODS**			
Database name	1	Name each individual database searched, stating the platform for each.	p 9, Appendix Text 3
Multi-database searching	2	If databases were searched simultaneously on a single platform, state the name of the platform, listing all of the databases searched.	n/a
Study registries	3	List any study registries searched.	n/a
Online resources and browsing	4	Describe any online or print source purposefully searched or browsed (e.g., tables of contents, print conference proceedings, web sites), and how this was done.	p 9
Citation searching	5	Indicate whether cited references or citing references were examined, and describe any methods used for locating cited/citing references (e.g., browsing reference lists, using a citation index, setting up email alerts for references citing included studies).	n/a
Contacts	6	Indicate whether additional studies or data were sought by contacting authors, experts, manufacturers, or others.	n/a
Other methods	7	Describe any additional information sources or search methods used.	p 9
**SEARCH STRATEGIES**			
Full search strategies	8	Include the search strategies for each database and information source, copied and pasted exactly as run.	Appendix Text 3
Limits and restrictions	9	Specify that no limits were used, or describe any limits or restrictions applied to a search (e.g., date or time period, language, study design) and provide justification for their use.	p 9, Appendix Text 3
Search filters	10	Indicate whether published search filters were used (as originally designed or modified), and if so, cite the filter(s) used.	n/a
Prior work	11	Indicate when search strategies from other literature reviews were adapted or reused for a substantive part or all of the search, citing the previous review(s).	n/a
Updates	12	Report the methods used to update the search(es) (e.g., rerunning searches, email alerts).	p 9
Dates of searches	13	For each search strategy, provide the date when the last search occurred.	p 9, Appendix Text 3
**PEER REVIEW**			
Peer review	14	Describe any search peer review process.	p 9
**MANAGING RECORDS**			
Total Records	15	Document the total number of records identified from each database and other information sources.	Appendix Text 3, Fig 1
Deduplication	16	Describe the processes and any software used to deduplicate records from multiple database searches and other information sources.	Appendix Text 3

PRISMA-S: An Extension to the PRISMA Statement for Reporting Literature Searches in Systematic Reviews
Rethlefsen ML, Kirtley S, Waffenschmidt S, Ayala AP, Moher D, Page MJ, Koffel JB, PRISMA-S Group.
Last updated February 27, 2020.

Supplementary Data 3: Search strategy details

Search 1. All searches run on 8/9/17. Date limits from 2005-2017. No language limits.

Database	Search strategy	Number of results
PubMed	(("Cell Phone"[MeSH] OR "Text Messaging"[MeSH] OR "Mobile Applications"[Mesh] OR "mobile health"[tw] OR "mobile application"[tw] OR "mobile applications"[tw] OR app[tw] OR apps[tw] OR "mobile phone"[tw] OR "mobile phones"[tw] OR messaging[tw] OR texting[tw] OR "text message"[tw] OR "text messages"[tw] OR "text messaging"[tw] OR text-message[tw] OR "instant messaging"[tw] OR "instant message"[tw] OR "instant messages"[tw] OR "mobile technology"[tw] OR "mobile technologies"[tw] OR mhealth[tw] OR m-health[tw] OR "cellular technology"[tw] OR "cellular technologies"[tw] OR "cellular phone"[tw] OR "cellular phones"[tw] OR "cell phone"[tw] OR "cell phones"[tw] OR cellphone[tw] OR cellphones[tw] OR "personal digital assistant"[tw] OR PDA[tw] OR smartphone[tw] OR smartphones[tw] OR "smart phone" OR "smart phones" OR SMS[tw] OR "short message"[tw] OR "short messages"[tw] OR "short messaging"[tw] OR handset[tw] OR handsets[tw] OR ehealth[tw] OR iphone[tw] OR iphones[tw] OR android[tw] OR "apple store"[tw] OR "apple app store"[tw] OR itunes[tw] OR "app store"[tw] OR "mobile communication"[tw] OR bluetooth[tw] OR handheld[tw] OR hand-held[tw] OR "patient portal"[tw] OR "portal use"[tw] OR "remote monitoring"[tw] OR telemonitor*[tw])<br><br>AND<br><br>("Hypertension"[MeSH] OR hypertension[tw] OR hypertensive[tw] OR "blood pressure"[tw]))	1470
Embase	('mobile phone'/exp OR 'text messaging'/exp OR 'mobile application'/exp OR "mobile health" OR app OR apps OR "mobile application" OR "mobile applications" OR "mobile phone" OR "mobile phones" OR messaging OR texting OR "text message" OR "text messages" OR "text messaging" OR text-message OR "instant messaging" OR "instant message" OR "instant messages" OR "mobile technology" OR "mobile technologies" OR mhealth OR m-health OR "cellular technology" OR "cellular technologies" OR "cellular phone" OR "cellular phones" OR "cell phone" OR "cell phones" OR cellphone OR cellphones OR "personal	4073

	digital assistant" OR PDA OR smartphone OR smartphones OR "smart phone" OR "smart phones" OR SMS OR "short message" OR "short messages" OR "short messaging" OR handset OR handsets OR ehealth OR iphone OR iphones OR android OR "apple store" OR "apple app store" OR itunes OR "app store" OR "mobile communication" OR bluetooth OR handheld OR hand-held OR "patient portal" OR "portal use" OR "remote monitoring" OR telemonitor*)<br><br>AND<br><br>('hypertension'/exp OR hypertension OR hypertensive OR "blood pressure")	
Web of Science	("mobile health" OR app OR apps OR "mobile application" OR "mobile applications" OR "mobile phone" OR "mobile phones" OR messaging OR texting OR "text message" OR "text messages" OR "text messaging" OR text-message OR "instant messaging" OR "instant message" OR "instant messages" OR "mobile technology" OR "mobile technologies" OR mhealth OR m-health OR "cellular technology" OR "cellular technologies" OR "cellular phone" OR "cellular phones" OR "cell phone" OR "cell phones" OR cellphone OR cellphones OR "personal digital assistant" OR PDA OR smartphone OR smartphones OR "smart phone" OR "smart phones" OR SMS OR "short message" OR "short messages" OR "short messaging" OR handset OR handsets OR ehealth OR iphone OR iphones OR android OR "apple store" OR "apple app store" OR itunes OR "app store" OR "mobile communication" OR bluetooth OR handheld OR hand-held OR "patient portal" OR "portal use" OR "remote monitoring" OR telemonitor*)<br><br>AND<br><br>(hypertension OR hypertensive OR "blood pressure")	2285
Google Scholar	(mhealth OR mobile OR "text message" OR telemonitor*) AND (hypertension OR hypertensive OR "blood pressure")	100
Total before duplicates		7928
Number of duplicates		2340
Total after de-duplication		5588

Search 2. Search update run on July 23, 2019. Date limits from 8/10/17 – 7/23/19. No language limits.

**Database**	**Search strategy**	**Number of results**
PubMed	See above	649
Embase	See above	1848

Web of Science	See above	1125
Google Scholar	See above	100
Total before duplicates		3722
Number of duplicates		1456
Total after de-duplication		2266

Supplementary Table 1: Studies that used theories or models to inform intervention design

Study	Behavior Theory/Model
Brewer, 2019	Health Belief Model, Social Cognitive Theory, Community Mobilization Model
Buis, 2017	Health Belief Model
Chow, 2015	Social Cognitive Theory, Theory of Planned Behavior, Theory of Reasoned Action, Operant Conditioning, Information-Motivation-Behavioral Skills Model, Control Theory
Davidson, 2015	Self-Determination Theory
Derose, 2019	Socioecological Theory
Gonzalez-Sanchez, 2019	Transtheoretical Model of Health Behavior Change
Kim MT et al, 2019	Information-Motivation-Behavioral Skills Model
Kim M, 2019	Interaction Model of Client Health Behavior
Levin, 2019	Attitude-Social Influence-Efficacy
Lewinski, 2019	Chronic Care Model, Health Decision Model, Transtheoretical Model of Health Behavior Change
Newton, 2018	Social Cognitive Theory
Orozco-Beltran, 2017	Chronic Care Model, Kaiser Permanente Model
Skolarus, 2017	Self-Determination Theory
Varleta, 2017	Social Cognitive Theory

Supplementary Table 2: GRADE Assessment

Study	Serious limitations related to bias	Serious limitations related to indirectness	Serious limitations related to imprecision	Overall Quality
Randomized Clinical Trials				
Alonso-Domínguez	None	BP not primary outcome (PA, adherence to Mediterranean diet); large portion of intervention not mHealth	None	Moderate
Buis	None	BP not primary outcome (med adherence); Insufficient follow-up length (1-mo)	wide confidence interval; intermediate sample size	Moderate-Low
Chow	None	BP not primary outcome (LDL)	None	Moderate
Davidson	Reporting bias (>20% not included in analysis)	None	Small sample size	Moderate[1]
Derose	Reporting bias (>20% not included in analysis)	Large portion of intervention not mHealth	Intermediate sample size; no confidence interval	Low-very low
Gonzalez-Sanchez	None	BP not primary outcome (CVD risk)	None	High-Moderate
Haufe	None	BP not primary outcome (Metabolic Syndrome Z score); large portion of intervention not mHealth	None	Moderate
Kim M	None	Large portion of intervention not mHealth; Insufficient follow-up length (8-wk)	wide confidence interval; intermediate sample size	Moderate-Low
McManus	None	None	None	High
Newton	Reporting bias (>20% not included in analysis)	BP not primary outcome (feasibility; weight loss); Large portion of intervention not mHealth	Small sample size	Very low
Or	None	Insufficient follow-up length (3-mo)	Small sample size	Moderate
Skolarus	Reporting bias (>20% not included in analysis)	BP not primary outcome (feasibility)	Small sample size	Very low
Varleta	Reporting bias (>20% not included in analysis)	BP not primary outcome (med adherence)	wide confidence interval; high sample size	Low
Wakefield	None	None	None	High
Zha	None	None	Small sample size	Moderate
Non-Randomized Clinical Trials				
Brewer	Observational study: cohort	None	Small sample size	Very low
Fukuoka	Observational study: cohort	BP not primary outcome (weight; BMI); Insufficient follow-up length (8-week)	Small sample size	Very low
Jones	Observational study: cohort	BP not primary outcome (feasibility); Insufficient follow-up length (12-wk)	Small sample size	Very low
Kim MT et al	Observational study: cohort	BP not primary outcome (feasibility)	None	Very low
Levin	Observational study: cohort	BP not primary outcome (med adherence); Insufficient follow-up length (12-wk)	Small sample size	Very low
Lewinski	Observational study: cohort	Large portion of intervention not mHealth	wide confidence interval; intermediate sample size	Very low
Milani	Observational study: matched cohort	Insufficient follow-up length (90-days)	wide confidence interval; intermediate sample size	Very low
Orozco-Beltran	Observational study: cohort	None	None	Low
Patel	Observational study: cohort	BP not primary outcome (med adherence)	Small sample size	Very low
Wenger	Observational study: cohort	BP not primary outcome (feasibility)	Small sample size	Very low

Abbreviations: BP (blood pressure); CVD (cardiovascular disease) LDL (low density lipoprotein); PA (physical activity)

[1]This study was increased from low to moderate due to large effect size

Supplementary Table 3: Data included in meta-regression analysis

Author	Year	Group	Baseline sample size	SBP at baseline Mean SBP (SD)	6-mo sample size	SBP at 6-mo Mean SBP (SD)
Zha	2019	Intervention	12	145.77 (5.1)	12	127.38 (4.86)
Zha	2019	Control	13	145.67 (3.68)	13	140.88 (5.01)
Wakefield	2011	Intervention	93	138 (19.733)	77	131.95 (20.464)
Wakefield	2011	Control	107	134 (16.712)	97	138.48 (17.426)
McManus	2018	Intervention	389	153.2 (14.3)	338	139 (16.8)
McManus	2018	Control	393	153.1 (14)	358	142.5 (15.4)
Skolarus	2017	Intervention	48	160.7 (23.6)	41	147.2 (21.8)
Skolarus	2017	Control	46	162.2 (20.5)	32	146.7 (20.5)
Newton	2019	Intervention	68	126.4 (14.3)	68	126.60 (23.91)
Newton	2019	Control	29	127.7 (14.1)	29	127.3 (3.9)
Haufe	2019	Intervention	160	138 (13)	160	132 (11)
Haufe	2019	Control	154	137 (14)	154	135 (12)
Chow	2015	Intervention	352	128.8 (12.3)	352	128 (14.36)
Chow	2015	Control	358	128.7 (12.2)	358	136 (14.48)

Abbreviations: SBP (systolic blood pressure); SD (standard deviation)

Supplementary Figure 1. Difference in mean systolic blood pressure between intervention and control group by population in (a) RCT studies or between the intervention group and the comparator group in (b) non-RCT studies

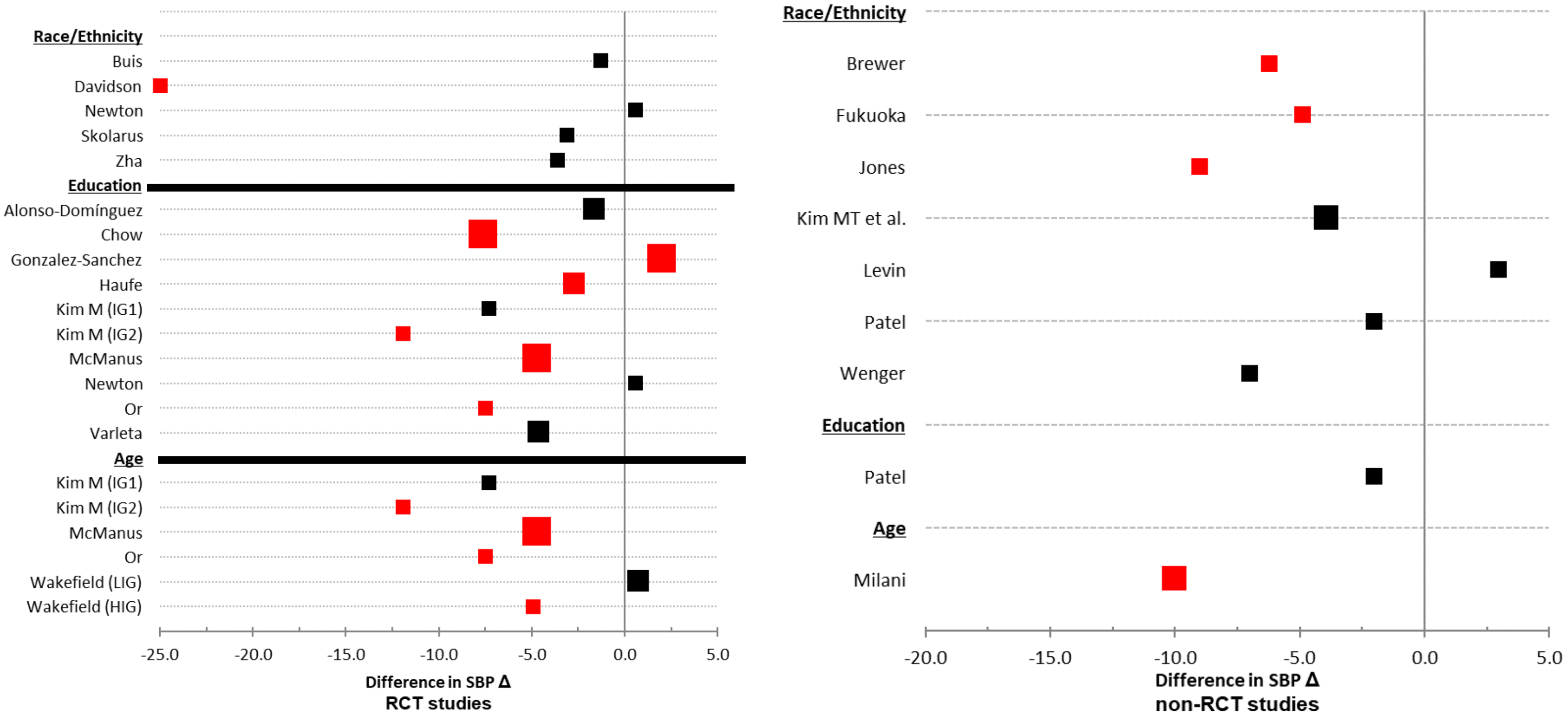

This figure shows the difference in mean systolic blood pressure change between the intervention group and the control group in RCT studies (left panel). For non-RCT studies, this figure shows the difference in mean SBP change for the intervention group and the comparison group (e.g., matched control, pre-intervention). The studies are grouped based on if they met inclusion for the review by race/ethnicity, educational attainment, or age criteria. Studies that met inclusion criteria based on multiple criteria are listed multiple times.

Abbreviations: SBP (systolic blood pressure); RCT (randomized clinical trial)
Red markers indicate statistically significant changes at $p < 0.05$.
Marker size correlates with sample size/precision based on GRADE checklist[75] criteria: low: <100 participants per experimental arm; intermediate: 100-300 participants per experimental arm; high: 300+ participants per experimental arm.
Derose (2019), an RCT that met inclusion criteria by race/ethnicity reported only a non-significant SBP change between the intervention and control group. (See Table 3)

Supplementary Figure 2. Difference in mean systolic blood pressure between intervention and control group by technology platform in (a) RCT studies or between the intervention group and the comparator group in (b) non-RCT studies

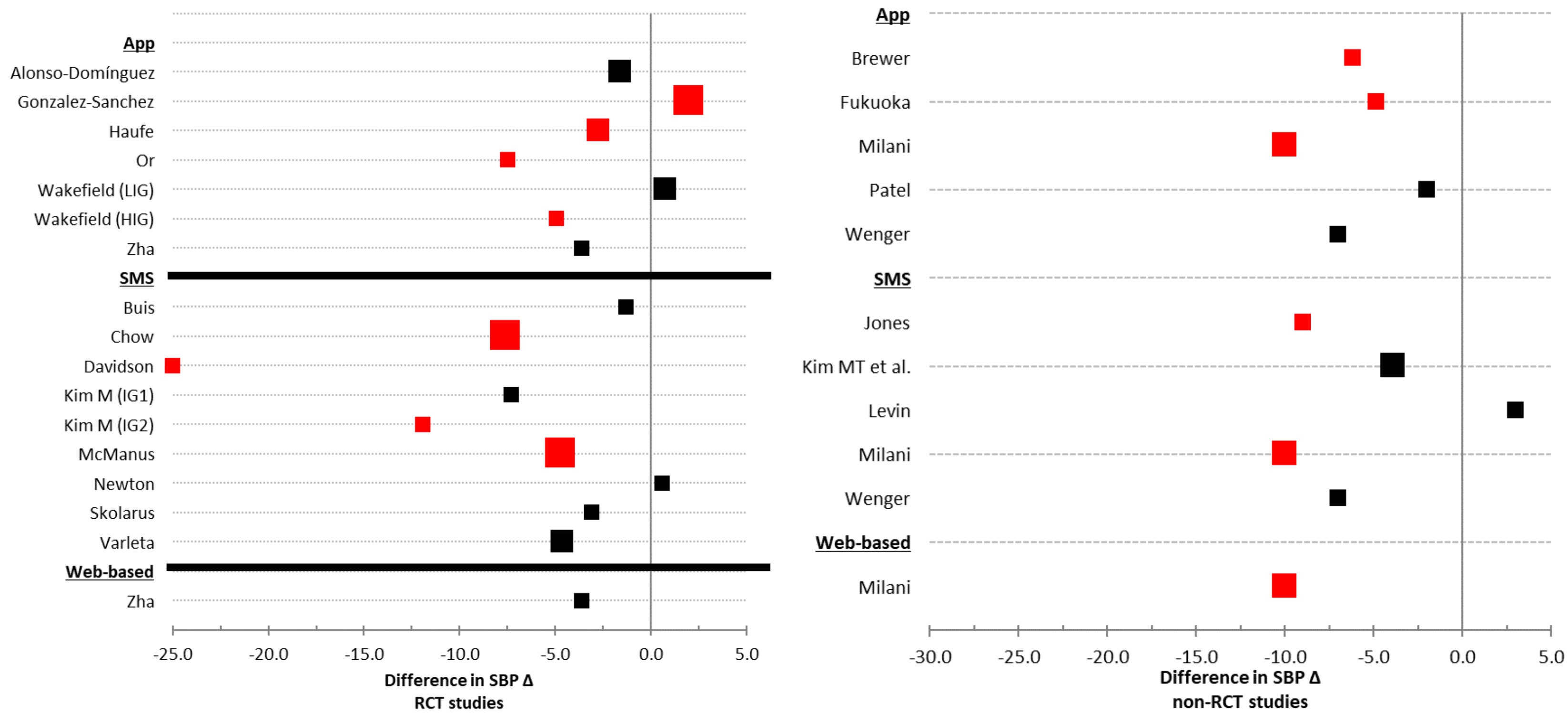

This figure shows the difference in mean systolic blood pressure change between the intervention group and the control group in RCT studies (left panel). For non-RCT studies, this figure shows the difference in mean SBP change for the intervention group and the comparison group (e.g., matched control, pre-intervention). The studies are grouped based on if the technology platform used was an app, text message (SMS), or web-based.

Abbreviations: SBP (systolic blood pressure); SMS (short message service, or text messaging); RCT (randomized clinical trial)
Red markers indicate statistically significant changes at $p < 0.05$.
Marker size correlates with sample size/precision based on GRADE checklist[75] criteria: low: <100 participants per experimental arm; intermediate: 100-300 participants per experimental arm; high: 300+ participants per experimental arm.
Derose (2019), an RCT that met inclusion criteria by race/ethnicity reported only a non-significant SBP change between the intervention and control group. (See Table 3)
