# Supplementary material for: Mobile health strategies for blood pressure self-management in urban populations with digital barriers: systematic review and meta-analyses
Source: NPJ Digit Med. 2021 Jul 22;4:114. doi: 10.1038/s41746-021-00486-5 (PMC8298448; doi:10.1038/s41746-021-00486-5)
Supplement: Supplementary file 2 — Reporting Summary [file 41746_2021_486_MOESM2_ESM.pdf]

nature research

Corresponding author(s): Elaine Khoong

Last updated by author(s): Mar 24, 2021

Reporting Summary

Nature Research wishes to improve the reproducibility of the work that we publish. This form provides structure for consistency and transparency in reporting. For further information on Nature Research policies, see our Editorial Policies and the Editorial Policy Checklist.

Statistics

For all statistical analyses, confirm that the following items are present in the figure legend, table legend, main text, or Methods section.

n/a	Confirmed	
☐	☒	The exact sample size (n) for each experimental group/condition, given as a discrete number and unit of measurement
☐	☒	A statement on whether measurements were taken from distinct samples or whether the same sample was measured repeatedly
☐	☒	The statistical test(s) used AND whether they are one- or two-sided<br>*Only common tests should be described solely by name; describe more complex techniques in the Methods section.*
☒	☐	A description of all covariates tested
☒	☐	A description of any assumptions or corrections, such as tests of normality and adjustment for multiple comparisons
☐	☒	A full description of the statistical parameters including central tendency (e.g. means) or other basic estimates (e.g. regression coefficient) AND variation (e.g. standard deviation) or associated estimates of uncertainty (e.g. confidence intervals)
☐	☒	For null hypothesis testing, the test statistic (e.g. F, t, r) with confidence intervals, effect sizes, degrees of freedom and P value noted<br>*Give P values as exact values whenever suitable.*
☒	☐	For Bayesian analysis, information on the choice of priors and Markov chain Monte Carlo settings
☒	☐	For hierarchical and complex designs, identification of the appropriate level for tests and full reporting of outcomes
☐	☒	Estimates of effect sizes (e.g. Cohen's d, Pearson's r), indicating how they were calculated

Our web collection on statistics for biologists contains articles on many of the points above.

Software and code

Policy information about availability of computer code

Data collection	N/A
Data analysis	N/A

For manuscripts utilizing custom algorithms or software that are central to the research but not yet described in published literature, software must be made available to editors and reviewers. We strongly encourage code deposition in a community repository (e.g. GitHub). See the Nature Research guidelines for submitting code & software for further information.

Data

Policy information about availability of data

All manuscripts must include a data availability statement. This statement should provide the following information, where applicable:
- Accession codes, unique identifiers, or web links for publicly available datasets
- A list of figures that have associated raw data
- A description of any restrictions on data availability

The data supporting the findings of this study are available within the paper and its supplementary information files.

Field-specific reporting

Behavioural & social sciences study design

All studies must disclose on these points even when the disclosure is negative.

Study description	Systematic review with meta-analysis
Research sample	Systematic review of studies focused on mobile health strategies for hypertension self-management among low-income, older, or minority populations
Sampling strategy	Inclusion criteria: Our review focuses on studies in urban populations in high-income countries, as defined by the World Bank, because barriers to care and the practice of medicine in rural areas and low-income countries are different. Within low and middle-income countries, the context of and infrastructure for digital care is notably different from high-income countries.71 In rural areas, which tend to have inadequate access to and supply of healthcare clinicians, mHealth interventions often focus on replacement of in-person clinical care rather than supplementing clinical care with self-management, which is the focus of this review.72 We defined "urban" according to the U.S. Census Bureau's definition for US locations only. Included studies had to focus on at least one population with known disparities in use of digital health tools, defined by any one of the following three characteristics: age (mean age >65 years); education (>60% high school education or less); and/or race/ethnicity (<50% non-Hispanic White for US studies). In addition, studies were included if their self-management interventions involved active engagement with a mobile technology such as a phone application, text messaging (short messaging service, or SMS), or a web-based platform. All studies were required to include blood pressure as an outcome.
Data collection	Data was collected from the published studies. Authors were contacted for additional data if needed
Timing	We conducted a systematic search for studies involving mHealth and hypertension from 2005-present in PubMed, Embase, Web of Science, and Google Scholar on August 9, 2017 and updated on July 23, 2019. We limited the search from 2005-2019 to reflect the fact that mobile phones were not widely used prior to 2005.
Data exclusions	Exclusion criteria: We excluded studies conducted in rural settings and non-English studies. For US locations, rural was defined by the US Census Bureau. Non-US locations were excluded if they self-identified as rural. We excluded studies that focused on pulmonary and gestational hypertension. In addition, studies were excluded if evaluations did not include disease management (e.g. usability of an app) or if no self-management intervention was described. We also excluded studies that did not measure blood pressure pre- and/or post-intervention. Studies without primary data (e.g. protocols, reviews) and studies without full text (e.g., unable to acquire full text and conference abstracts only) were excluded.
Non-participation	N/A
Randomization	N/A

Reporting for specific materials, systems and methods

We require information from authors about some types of materials, experimental systems and methods used in many studies. Here, indicate whether each material, system or method listed is relevant to your study. If you are not sure if a list item applies to your research, read the appropriate section before selecting a response.

Materials & experimental systems

n/a	Involved in the study	
☒	☐	Antibodies
☒	☐	Eukaryotic cell lines
☒	☐	Palaeontology and archaeology
☒	☐	Animals and other organisms
☒	☐	Human research participants
☒	☐	Clinical data
☒	☐	Dual use research of concern

Methods

n/a	Involved in the study	
☒	☐	ChIP-seq
☒	☐	Flow cytometry
☒	☐	MRI-based neuroimaging
